# Hypomethylation of the *DAZ3* promoter in idiopathic asthenospermia: a screening tool for liquid biopsy

**DOI:** 10.1038/s41598-020-75110-9

**Published:** 2020-10-22

**Authors:** Shichang Zhang, Li Xu, Mengyao Yu, Jiexin Zhang

**Affiliations:** 1 Department of Laboratory Medicine, The First Affiliated Hospital of Nanjing Medical University, Nanjing, 210029 China; 2 Department of Clinical Nutrition, The First Affiliated Hospital of Nanjing Medical University, Nanjing, 210029 China

**Keywords:** Reproductive disorders, Genetic markers

## Abstract

Given the role of the *deleted in azoospermia* gene in male infertility, whether the somatic *deleted in azoospermia* methylation status is associated with idiopathic asthenospermia should be determined. To investigate the methylation levels of the *deleted in azoospermia* promoter in peripheral white blood cells from idiopathic asthenospermia patients relative to those in normozoospermia controls, 61 ethylene diamine tetraacetic acid anticoagulant blood samples were drawn from all participants for DNA isolation. The *deleted in azoospermia* promoter methylation ratio was detected by MassARRAY-based methylation quantification and confirmed by quantitative methylation-specific polymerase chain reaction. A MassARRAY-based methylation analysis showed that the *deleted in azoospermia 3* promoter (0 to − 2 kbp) was significantly hypomethylated in peripheral white blood cells from idiopathic asthenospermia males, specifically one CpG site (− 246 to − 247). Quantitative methylation-specific polymerase chain reaction data further confirmed that the methylation level of the *deleted in azoospermia 3* promoter region in idiopathic asthenospermia patients was significantly lower than that in normozoospermia males. The area under the receiver operating characteristic curve determined by quantitative methylation-specific polymerase chain reaction was 0.737 (95% confidence interval: 0.552 to 0.924), with a sensitivity of 53.9% and a specificity of 88.2% at a cut-off level of 74.7%. Therefore, our results suggested that methylation ratio detection of the *deleted in azoospermia 3* promoter region by real-time polymerase chain reaction assay is a promising and feasible tool for liquid biopsy in the clinical laboratories. The methylation status of other reported infertility-related genes should also be investigated in peripheral white blood cells.

## Introduction

To date, considerable attention has been paid to infertility caused by asthenospermia. Idiopathic asthenospermia (IAS) falls into this category but has an unknown etiology. IAS males have normal sperm parameters except for low sperm motility^1,2^. Karyotype analysis is an important technique for chromosome examination and genetic background screening. However, most subfertile patients do not have an abnormal karyotype, which is consistent with our unpublished results showing that 343 of 357 peripheral lymphocytes from subfertile men were normal using G-banding-based karyotype screening over the past 10 years. These data indicate that IAS with a “clean karyotype background” is one of the leading causes of male infertility^3^. Therefore, subtle genetic defects in the somatic cells of IAS males with a clean karyotype background, which may be missed by G-banding screening, need to be further investigated^4^.

Several genes determine spermatogenesis^5,6^. The *deleted in azoospermia* (*DAZ*) gene, which was identified by a Y chromosome-specific DNA probe^7^, has been detected in the *azoospermia factor* (*AZF)* region c, where microdeletions typically occur^8^. *DAZ* is expressed in premeiotic germ cells (especially spermatogonia)^9,10^. Numerous reports have shown that sperm *DAZ* microdeletion/gene copy loss reduces DAZ protein expression in the testis, inhibits spermatogenesis, and finally causes germ cell degeneration^11,12^. Mozdarani et al*.* described a combined primed in situ labeling (PRINS) and fluorescence in situ hybridization (FISH) technique in sperm nuclei for practical *DAZ* screening^13^. Currently, sperm microdeletion detection is recommended guideline for male infertility^14,15^. One report demonstrated that the *DAZ* deletion in spermatozoa also occurs in the somatic cells of fathered sons, indicating that subtle genetic changes in *DAZ* in sperm are somehow relevant to its status in somatic cells^16^. Nevertheless, DNA methylation is another important change that regulates embryonic development through epigenetic modification. DNA methylation is the catalytic transfer of a methyl group from an active methyl-containing compound, such as S-adenosylmethionine, to DNA, and it generally occurs at CpG sites in vertebrates. In a genome-wide study, a group of 2752 aberrantly methylated CpGs in subfertile sperm was identified via customized arrays^17^. However, detailed research has not been performed on the somatic *DAZ* methylation status in IAS to date.

In this study, peripheral white blood cell (pWBCs) samples from IAS males who had passed the G-banding examination were collected. *DAZ* promoter methylation was detected by a MassARRAY-based quantification assay and further verified by quantitative methylation-specific PCR (Q-MSP).

## Results

### Study design and patient cohort

We recruited 61 patients who visited our hospital for fertility counseling. According to the exclusion criteria, there were 26 IAS males and 35 age-matched normozoospermia (NZ) males with confirmed healthy offspring. Their main characteristics are listed in Table 1. Age, sperm concentration and serum hormone levels showed no differences between groups, and only sperm motility was significantly decreased in the IAS group.

**Table 1:** Sperm parameters and characterization of the analyzed populations.

Groups	NZ (n = 35)	IAS (n = 26)	*P* value
Age (years)	29.0 (26.0, 33.8)	31.0 (28.0, 35.5)	0.1033
Sperm			
PM (%)	37.20 (33.80, 45.50)	13.80 4.28, 18.70)	< 0.0001***
TM (%)	65.40 (60.10, 70.40)	29.25 (16.03, 36.20)	< 0.0001***
Concentration (× 10^6^/mL)	85.00 (54.90, 116.70)	65.15 (46.93, 114.8)	0.2409
Hormone			
Estradiol (pmol/L)	115.0 (96.0, 143.0)	117.5 (102.3, 143.5)	0.9593
FSH (IU/L)	4.42 (3.15, 6.26)	4.80 (4.56, 6.25)	0.2074
LH (IU/L)	3.58 (2.64, 4.67)	3.34 (2.45, 4.14)	0.3984
Prolactin (mIU/L)	185.7 (138.1, 12.1)	189.3 (125.8, 222.5)	0.6379
Progesterone (nmol/L)	1.75 (1.46, 2.42)	1.88 (1.70, 2.21)	0.3438
Testosterone (nmol/L)	13.23 (10.55, 15.53)	13.38 (10.49, 17.69)	0.3963

Data were presented as the medians (interquartile range).

PM, progressive motility; TM, total motility; FSH, follicle stimulating hormone; LH, luteinizing hormone.

***P < 0.001.

### Hypomethylated sites in the DAZ promoter in pWBCs from IAS males

**Figure 1:**
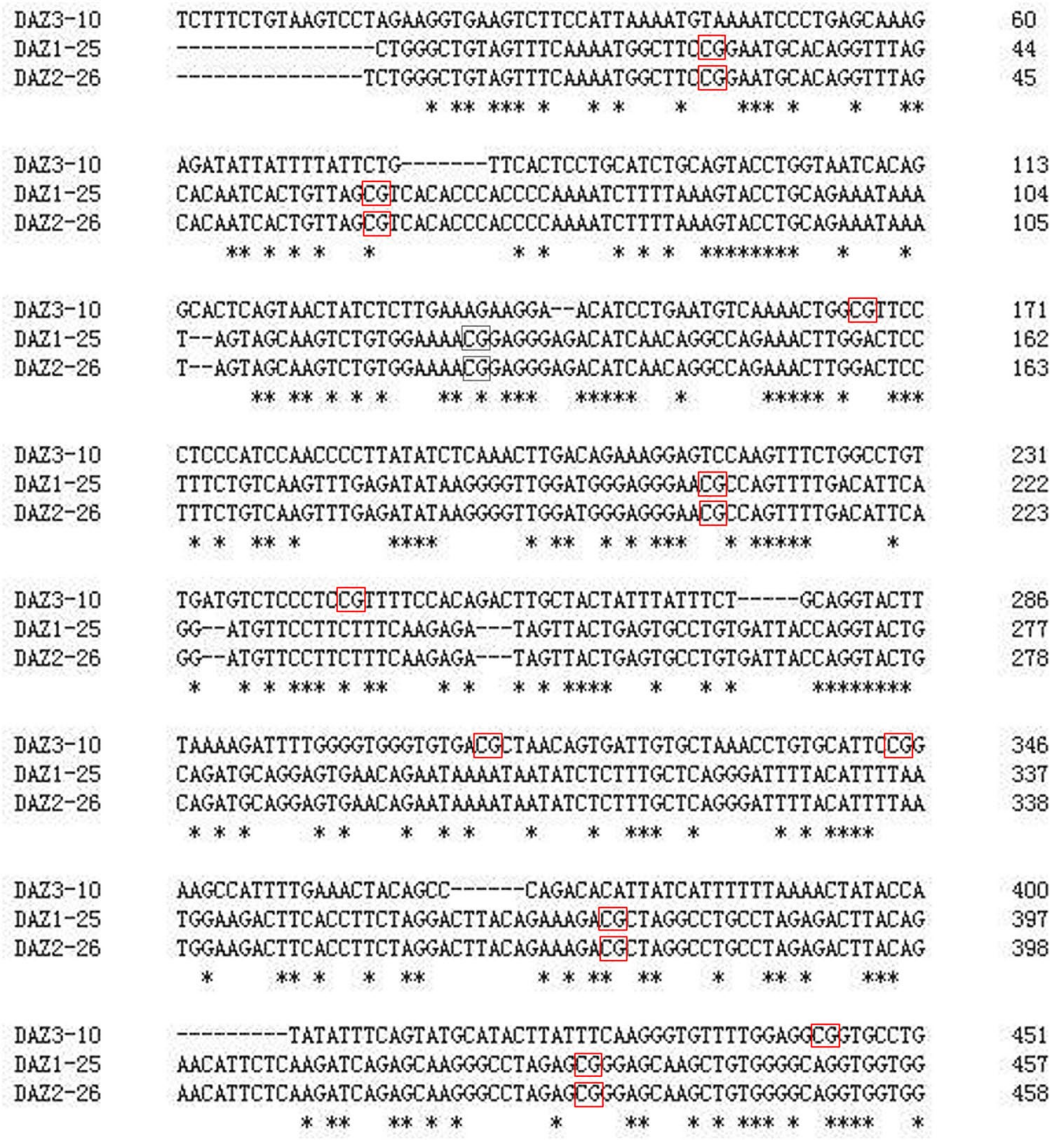
Distribution of 15 detectable CpG sites via the MassARRAY platform. Notably, each marked CpG site in the *DAZ2* promoter region is a duplicate of a CpG site from an identical location in the *DAZ3* promoter that is of the same mass.

**Figure 2:**
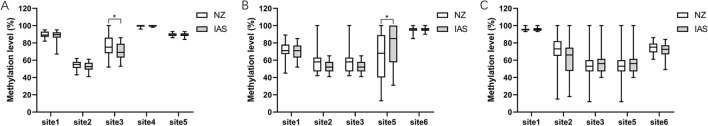
Methylation levels of each CpG site in the *DAZ* promoters. Mean methylation levels of each CpG site in the (**A**) *DAZ3* promoter, (**B**) *DAZ1* promoter, and (**C**) *DAZ2* promoter. Data are presented as the medians (interquartile ranges) (*P < 0.05).

Based on a primer design principle that covered candidate CpG sites as much as possible, PCR products were intermittently and evenly distributed in the *DAZ* promoter region (− 2 k bp from the transcription start site; Fig. 1). The methylation ratio of each CpG site was quantitatively measured via the MassARRAY platform in all 61 pWBC samples. The results showed a significantly reduced methylation percentage at the 3rd CpG site (− 246 to − 247) in the *DAZ3* promoter of IAS pWBCs when compared with that of the NZ controls [69.0% (63.0%, 79.0%) vs. 75.0% (68.0%, 86.0%), *P* = 0.0429; Fig. 2A]. The methylation level at the 5th CpG site (− 261 to − 262) in the *DAZ1* promoter was also significantly different in IAS pWBCs compared with that in the NZ controls [85.0% (57.5%, 100.0%) vs. 68% (40.0%, 100.0%), *P* = 0.0233; Fig. 2B]. However, differences in methylation level were not observed between IAS and NZ males at other CpG sites in the *DAZ* promoter (Fig. 2A-C).

### DAZ3 promoter hypomethylation in IAS patients

**Figure 3:**
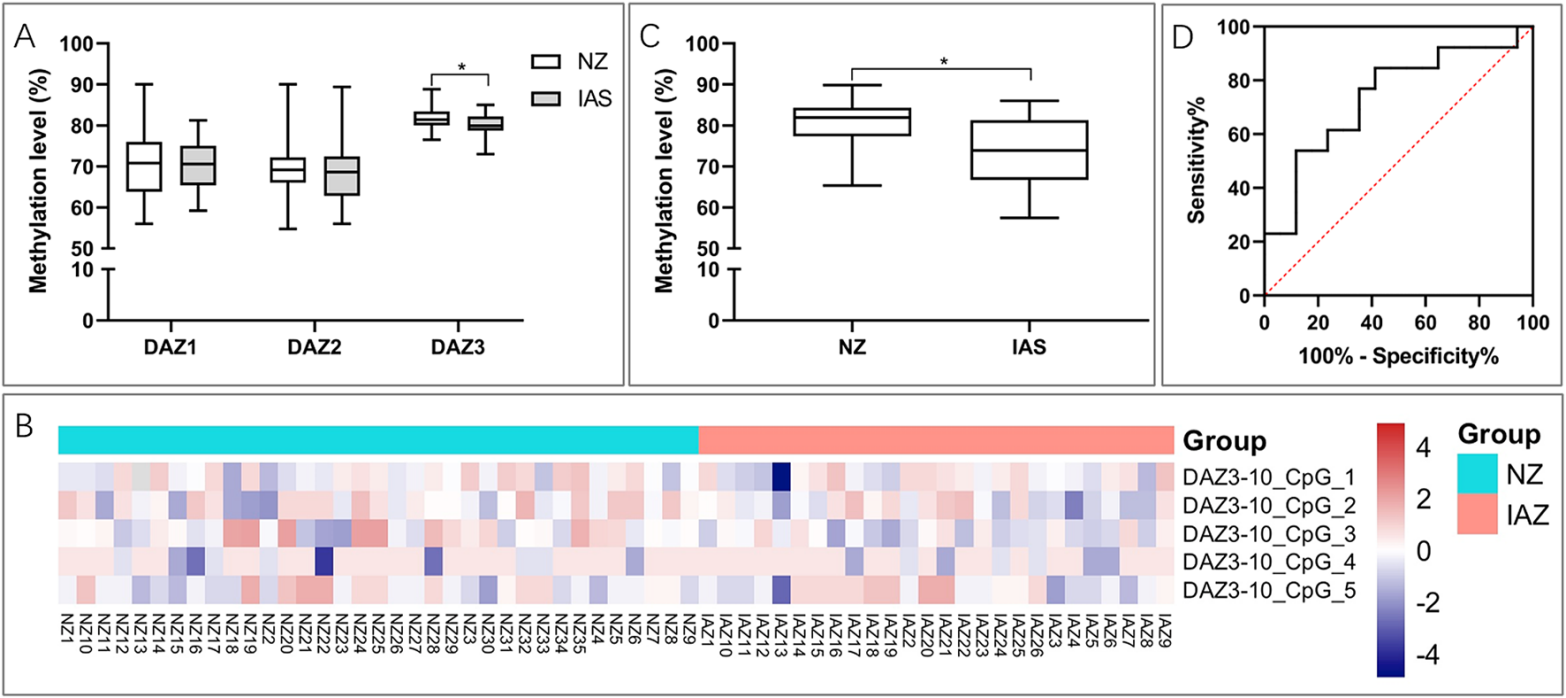
Methylation levels of the *DAZ3* promoter in IAS and NZ males. (**A**) Methylation levels of the *DAZ3* promoter (0 to − 2 kbp) calculated via the MassARRAY platform. Data are presented as the medians (interquartile ranges) (*P < 0.05). (**B**) Two-way hierarchical cluster analysis of IAS and NZ (columns) and DNA methylation of five CpG sites in the *DAZ3* promoter regions (rows). (**C**) *DAZ3* promoter (0 to − 2 kbp) methylation levels calculated via Q-MSP in 13 IAS patients and 17 NZ controls. Data are presented as the means ± SD (*P < 0.05). (**D**) Receiver operating characteristic (ROC) curves for the methylation levels of the *DAZ3* promoter, which were determined by Q-MSP.

Next, we averaged the methylation ratios of five CpG sites in each *DAZ* promoter to represent the overall promoter methylation percentage in each pWBC sample and compared IAS to NZ. Only the *DAZ3* promotor was significantly hypomethylated in IAS patients [79.9% (78.8%, 82.2%) vs. 81.4% (80.0%, 83.4%), *P* = 0.0346; Fig. 3A-B]. We applied Q-MSP to the validation cohort for further verification. The methylation level of the *DAZ3* promoter in the IAS pWBCs was significantly lower than that in the NZ pWBCs [73.9% (66.7%, 81.3%) vs. 81.9% (77.3%, 84.3%), *P* = 0.0279; Fig. 3C], which confirmed the results from the Sequenom MassARRAY platform. Receiver operating characteristic (ROC) curve analysis showed that the area under the curve (AUC) value was 0.737 (95% CI: 0.552 to 0.924). When the Youden index was the maximum value, the methylation level of *DAZ3* demonstrated an optimum diagnostic sensitivity of 53.9% and specificity of 88.2% at a cut-off level of 74.7% (Fig. 3D).

## Discussion

Our study demonstrated for the first time that the *DAZ3* promoter is significantly hypomethylated in IAS pWBCs compared with that in NZ pWBCs, especially the CpG site at − 246 to − 247. Our data further indicated that this epigenetic alteration can be detected and differentiated via Q-MSP method in the clinical laboratories, suggesting that (1) peripheral blood samples may be a secondary accessible source for IAS liquid biopsy; and that (2) both the hypomethylated region and the matched Q-MSP method will fill the detection gap in IAS pWBCs and can be considered as a promising supplementary to traditional karyotype analysis.

The first study elaborating on the DNA methylation pattern of the *DAZ* promoter was performed in sperm populations^18^. Arguments have been made about whether the failure of *DAZ* in somatic cells represents its role in germ cells; moreover, PCR assays may not be suitable for infertility diagnosis in pWBCs. However, a thorough study by Friemel et al*.* discovered a total of 471 differentially methylated CpGs in the peripheral blood of infertile men, and they proposed two surrogate DNA methylation markers, PIWIL1 and PIWIL2^19^. However, “*DAZ*” is not included in his online open list, which is most likely due to the use of BeadChip. Compared to this kind of defined chip, the MassARRAY platform we used in this study is suitable for quantitative detection of the methylation ratio at a designated CpG site or multiple CpG sites. This platform is sensitive enough to distinguish subtle methylation changes of only 5% and thus was adequate for detecting the variations in our study. Moreover, in contrast to the situation in the three previously cited studies^17,18,20^, cases of azoospermia, oligozoospermia or sperm deformity as well as abnormal serum hormone levels or spermophlebectasia, were deliberately excluded in our study. Based on the selected criteria, 26 IAS males who had a normal karyotype and 35 NZ males who had healthy offspring were assessed, and the methylation levels of 15 CpG sites in three *DAZ* promoters were compared. We provided additional data for the methylation study in IAS pWBCs.

Experiments conducted in murine models have shown that the expression of DAZ-like is induced after selective DNA demethylation of germ cell differentiation genes and its activation is central to the establishment of the germline genetic profile^21–23^. Therefore, hypermethylation of the *DAZ* gene may damage sperm quality. Sperm arise from somatic cell spermatogonia. During this process, sperm progenitors experience germline-specific epigenetic reprogramming firstly via comprehensive DNA demethylation followed by selective methyl addition for remodeling^24,25^. In other words, regardless of the methylation status of the *DAZ3* promoter in somatic cells, it would most likely be overwhelmed in the sperm. Therefore, hypermethylation of the *DAZ* gene places a greater emphasis on dysfunctional methylation in the testicular microenvironment. In contrast, the relatively hypomethylated *DAZ3* in pWBCs might reflect another unidentified epigenetic modulation mechanism during embryonic development from germ cells to somatic cells.

Numerous factors cause asthenospermia, including genetic defects, immune dysfunction, reproductive tract infection, and varicocele, among which genetic defects have the greatest importance. Fertility-related abnormal karyotypes not only lead to defective structures of reproductive organs but also affect spermatogenesis and thereby infertility. This is one of the typical symptoms of Klinefelter syndrome with a 47 XXY karyotype, which is a result of segregation failure of the X chromosome during meiosis. However, we suggest that subclinical genetic defects, such as methylation alternations, are far more important and deserve much more attention.

Liquid biopsy has better clinical application prospects and is more acceptable to patients. Based on pWBCs, methylation ratio detection by real-time PCR assay is very convenient for laboratory detection. However, due to the limited number of specimens analyzed in this study, further clinical evaluation of the sensitivity and specificity of this methodology is needed.

In summary, hypomethylation of the *DAZ3* promoter in pWBCs is a potential indicator for IAS. This preliminary study provides new insights into genetic screening for asthenospermia with the help of liquid biopsy.

## Materials and methods

### Exclusion criteria and sample collection

We collected ethylene diamine tetraacetic acid (EDTA) anticoagulant blood samples from subfertile males who had been diagnosed with a normal karyotype via G-banding examination. Detailed information, including the results of sperm examination, serum hormone tests and testicle ultrasound, were documented at each visit. Patients with azoospermia, oligozoospermia or sperm deformity as well as abnormal serum hormone levels or spermophlebectasia were excluded. The EDTA anticoagulant blood samples from 61 men were divided into two groups: normozoospermia (NZ) controls (n = 35) and IAS patients (n = 26). All 61 pWBCs were detected by MassARRAY-based methylation quantification. The 13 IAS patients and 17 NZ controls, which were derived from 61 patients were determined by Q-MSP. This research was performed in compliance with the Helsinki Declaration and authorized by the Ethical Committee of the First Affiliated Hospital of Nanjing Medical University (Nanjing, China). All patients signed an informed consent form agreeing to supply information and samples.

### DNA extraction

Genomic DNA from peripheral blood samples was extracted using a DNA Blood Mini Kit (Qiagen, Germany) according to the manufacturer’s instructions. The concentration and purity of the DNA were determined by absorbance at 260 and 280 nm and by agarose electrophoresis. The total amount of DNA was at least 2 µg.

### Primer design

Polymerase chain reaction (PCR) primers used in the Sequenom MassARRAY platform were designed with Epidesigner (https://epidesigner.com). For each reverse primer, an additional T7-promoter tag for in vitro transcription was added as well as a 10-mer tag on the forward primer to adjust for melting temperature differences. Primers used in Q-MSP were designed by Primer 5 software. The target regions were amplified using the primer pairs shown in Tables 2–3.

**Table 2:** Primers for genomic DNA methylation analysis via MassARRAY.

Application	Name	Left primer	Direction
MassARRAY	DAZ1-F	TTGGGTTGTAGTTTTAAAATGGTTT	Reverse
	DAZ1-R	CCAAATACTAAAATAATCCCCAAAA	
	DAZ2-F	TTTGGGTTGTAGTTTTAAAATGGTT	Reverse
	DAZ2-R	TATAATTAACTCCACCACCTACCCC	
	DAZ3-F	TTTTTTTGTAAGTTTTAGAAGGTGAA	Forward
	DAZ3-R	CCAATACCTAAATTCCAAACAAACA

**Table 3:** Primers for genomic DNA methylation analysis via Q-MSP.

Application	Name	Left primer
Q-MSP	DAZ3-S1-MF	AATATTTTGAATGTTAAAATTGGC
	DAZ3-S1-MR	CATCAACAAACCAAAAACTTAAAC
	DAZ3-S1-UF	GAATATTTTGAATGTTAAAATTGGT
	DAZ3-S1-UR	CATCAACAAACCAAAAACTTAAAC
	DAZ3-S2-MF	AATATTTTGAATGTTAAAATTGG
	DAZ3-S2-MR	AATAATAACAAATCTATAAAAAACG
	DAZ3-S2-UF	AATATTTTGAATGTTAAAATTGG
	DAZ3-S2-UR	AAATAATAACAAATCTATAAAAAACA
	DAZ3-S3-MF	GTTTAAGTTTTTGGTTTGTTGATG
	DAZ3-S3-MR	CAAATTTAACACAATCACTATTAACG
	DAZ3-S3-UF	GTTTAAGTTTTTGGTTTGTTGATG
	DAZ3-S3-UR	CAAATTTAACACAATCACTATTAACA
	DAZ3-S4-MF	GTTTAAGTTTTTGGTTTGTTGATG
	DAZ3-S4-MR	ACTATAATTTCAAAATAACTTCCG
	DAZ3-S4-UF	GTTTAAGTTTTTGGTTTGTTGATG
	DAZ3-S4-UR	AACTATAATTTCAAAATAACTTCCA

MF, methylated forward primer; UF, unmethylated forward primer; MR, methylated reverse primer; UR, unmethylated reverse primer.

### DNA pretreatment

A total of 1.5 µg of genomic DNA from each sample was bisulfite-treated with the an EZ DNA Methylation-Gold kit (Zymo Research, USA). The quality of the bisulfite conversion was controlled by using PCR products that had no methyl group. Bisulfite-treated DNA was amplified by a PCR Accessory Set (Sequenom, USA) followed by shrimp alkaline phosphatase (SAP) treatment, in vitro transcription (IVT) and RNase digestion by the MassCLEAVE Kit (Sequenom).

### Quantitative methylation analysis

The Sequenom MassARRAY platform (CapitalBio, China) was used to perform quantitative methylation analysis of *DAZ1*-*3* (GenBank accession number NC_000024.10). Detailed detection mechanisms were interpreted as described previously^26^. PCR amplification was performed as follows: a hot start at 94 °C for 15 min; 45 cycles of denaturation at 94 °C for 20 s, annealing at 56 °C for 30 s, and extension at 72 °C for 1 min and a final incubation at 72 °C for 3 min. Then, 2 ml of premix including 0.3 U of SAP (Sequenom), was added to dephosphorylate the unincorporated dNTPs. The reaction mixture was incubated at 37 °C for 40 min. SAP was inactivated for 5 min at 85 °C before further treatment. The PCR mixture was used as a template for IVT, and RNase A cleavage was used as the reverse reaction. The mixture was conditioned and spotted on a 384-pad Spectro-CHIP (Sequenom) by a MassARRAY nanodispenser (Samsung, USA), followed by spectral acquisition on a MassARRAY Compact MALDI-TOF (Sequenom). Five CpG sites were examined in each *DAZ* member (15 CpG sites in total). The methylation ratios were analyzed by EpiTyper software v1.0 (Sequenom, https://www.epidesigner.com/) to generate quantitative results for each CpG site or an aggregate of multiple CpG sites.

### Q-MSP DNA methylation analysis

The samples of 13 IAS patients and 17 NZ controls from 61 men were determined by Q-MSP, which was performed using SYBR Green Real-time PCR Master Mix (TOYOBO, Japan) and detected by a CFX96 real-time PCR system (Bio-Rad, USA). We used a no-template control (NTC), whole methylated genomic DNA and genomic DNA without bisulfite conversion as templates (QIAGEN, 59,568) to verify the Q-MSP system. According to the recommended method and equation^27^, we calculated the DNA methylation ratio via C_meth_ = 100/[1 + 2^(CTCG − CTTG)^]%.

### Statistical analysis

Data are expressed as medians and interquartile ranges (25th, 75th percentiles). Statistical analyses were performed using GraphPad Prism 8.0 (GraphPad, San Diego, CA, USA, https://www.graphpad.com/scientific-software/prism/). Nonparametric Mann–Whitney tests were used to analyze differences in methylation levels between the two groups. Receiver operating characteristic (ROC) curves and area under the curve (AUC) values were calculated for the distribution of methylation levels in IAS versus NZ, and 95% confidence intervals (CIs) were reported. According to the ROC curve, we calculated to determine the largest Youden index and found the corresponding ideal cut-off for the predicted risk value. A value of *P* < 0.05 was considered statistically significant.

## Data Availability

All data generated or analyzed during this study are included in this published article (and its Supplementary Information Files).
